# Inhibition of eIF2α dephosphorylation accelerates pterostilbene-induced cell death in human hepatocellular carcinoma cells in an ER stress and autophagy-dependent manner

**DOI:** 10.1038/s41419-019-1639-5

**Published:** 2019-05-28

**Authors:** Chen-Lin Yu, Shun-Fa Yang, Tung-Wei Hung, Chia-Liang Lin, Yi-Hsien Hsieh, Hui-Ling Chiou

**Affiliations:** 1 Institute of Medicine, Chung Shan Medical University, Taichung, Taiwan; 2 Department of Medical Research, Chung Shan Medical University Hospital, Taichung, Taiwan; 3 Division of Nephrology, Department of Medicine, Chung Shan Medical University Hospital, Taichung, Taiwan; 4 Institute of Biochemistry, Microbiology and Immunology, Chung Shan Medical University, Taichung, Taiwan; 5 Department of Biochemistry, School of Medicine, Chung Shan Medical University, Taichung, Taiwan; 6 Clinical laboratory, Chung Shan Medical University Hospital, Taichung, Taiwan; 7 School of Medical Laboratory and Biotechnology, Chung Shan Medical University, Taichung, Taiwan; 8 Department of Clinical Laboratory, Chung Shan Medical University Hospital, Taichung, Taiwan

**Keywords:** Toxicology, Liver cancer

## Abstract

Hepatocellular carcinoma (HCC) is the one of the most common cancers worldwide. Because the side effects of current treatments are severe, new effective therapeutic strategies are urgently required. Pterostilbene (PT), a natural analogue of resveratrol, has diverse pharmacologic activities, including antioxidative, anti-inflammatory and antiproliferative activities. Here we demonstrated that PT inhibits HCC cell growth without the induction of apoptosis in an endoplasmic reticulum (ER) stress- and autophagy-dependent manner. Mechanistic studies indicated that the combination of salubrinal and PT modulates ER stress-related autophagy through the phospho-eukaryotic initiation factor 2α/activating transcription factor-4/LC3 pathway, leading to a further inhibition of eIF2α dephosphorylation and the potentiation of cell death. An in vivo xenograft analysis revealed that PT significantly reduced tumour growth in mice with a SK-Hep-1 tumour xenograft. Taken together, our results yield novel insights into the pivotal roles of PT in ER stress- and autophagy-dependent cell death in HCC cells.

## Introduction

Hepatocellular carcinoma (HCC) is the most common type of primary liver cancer and the third leading cause of cancer mortality worldwide^1^. The mortality of HCC is high because of potential curative treatments being effective only at the early disease stages^2^ and drug resistance being developed. Therefore, novel effective therapeutic strategies are urgently required.

Pterostilbene (PT, *trans*-3,5-dimethoxy-4′-hydroxystilbene), a natural dimethylated analogue of resveratrol, is produced by Pterocarpus plants, *Vitis vinifera* leaves and grapes, and some berries^3^. PT exhibits various pharmacologic activities, including anti-inflammatory, antioxidative and antiproliferative activities^4^. Moreover, PT exhibits toxicity to cancer cells of various origins, including lung, prostate and colon^5–7^. Although PT can inhibit the HCC cell invasion and migration^8^, the mechanism underlying its cytotoxicity to HCC cells and the role of autophagy remain unclear.

Autophagy is a critical intracellular degradation mechanism responsible for trafficking aggregated proteins, damaged organelles and other undesirable cytoplasmic materials for lysosomal degradation under cellular stress^9^. Autophagy is a mechanism for cellular survival in periods of cellular stress; however, it can also lead to programmed cell death-II under certain conditions^10^. The endoplasmic reticulum (ER) is a perinuclear organelle responsible for Ca^2+^ storage, proteins and lipid synthesis, and protein modification and folding. Alteration of ER homeostasis leads to the accumulation of unfolded proteins in the ER lumen, leading to ER stress and unfolded protein response (UPR) pathway activation^11^. Furthermore, PT attenuates cell growth through ER stress induction^12^. In the presence of a misfolded protein, GRP78 is released from the ER transmembrane receptor inositol-requiring enzyme 1, thereby activating protein kinase RNA-like ER kinase (PERK) and activating transcription factor-6 (ATF-6). This in turn activates UPR signalling to increase the ER capacity. However, when ER stress is prolonged, the UPR pathway can also induce cell death^13^.

Eukaryotic initiation factor 2α (eIF2α) is a downstream effector of the UPR and a key initiator of messenger RNA translation under normal conditions^14^. In response to ER stress, the PERK-induced phosphorylation of eIF2α suppresses gene translation and enhances the expression of genes containing a short upstream open reading frame^15^. ATF4 is one of these genes with enhanced expression; the increased expression of ATF4 increases its target genes related to apoptosis and autophagy^16^. In response to ER stress, autophagy is also activated by the PERK pathway to facilitate the clearance of misfolded proteins^17^ or promote cell death^18^. Therefore, we investigated whether PT induces autophagic cell death through ER stress-signalling pathways in HCC cells.

## Materials and methods

### Chemicals and reagents

PT (purity ≥ 98%) and 3-methyladenine (3-MA) were purchased from Enzo Life Sciences (Farmingdale, NY, USA). Chloroquine (CQ), 3-(4,5-dimethylthiazol-2-yl)-2,5-diphenyl-tetrazolium bromide (MTT) and 4-phenylbutyric acid were purchased from Sigma-Aldrich (St. Louis, MO, USA). Antibodies for p62 and LC3 were purchased from Novus Biologicals (Littleton, CO, USA), and antibodies for cleaved-caspase-3, cleaved-poly (ADP-ribose) polymerase (PARP), Bip, PERK, eIF2α, phospho-eIF2α, ATF4, calreticulin and CHOP (C/EBP homologous protein) were purchased from Cell Signaling Technology (Danvers, MA, USA). Antibodies for Beclin-1, lamin B, α-tubulin, salubrinal (Sal), E-64d and pepstatin A (lysosomal protease inhibitors), small interfering RNA (siRNA)-eIF2α (si-eIF2α) and siRNA-LC3 (si-LC3) were purchased from Santa Cruz Biotechnology (Santa Cruz, CA, USA).

### Cell culture

HCC cell lines Huh-7, SK-Hep-1, PLC/PRF/5, HA22T/VGH and HepG2 were cultured in Dulbecco’s modified Eagle’s medium or minimum essential medium (Gibco BRL, Carlsbad, CA, USA) supplemented with 10% foetal bovine serum (Gibco BRL, Rockville, MD, USA) at 37 °C in a humidified atmosphere containing 5% CO_2_.

### Cell cytotoxicity assay

For the cell cytotoxicity assay, 4 × 10^4^ cells/well were seeded in 24-well plates and treated with various concentrations of PT (0, 25, 50, 75 and 100 μM) for 24 or 48 h. MTT was added to each well at a final concentration of 0.5 mg/ml, and the cells were incubated for an additional 4 h. The viable cells were directly proportional to the amount of formazan produced; formazan is a reduction product of MTT from dehydrogenases in the mitochondria. Colour intensity was measured at 570 nm after formazan was dissolved in methanol.

### Cell viability assay

The effect of PT on cell viability was assayed using the trypan blue dye exclusion method. HCC cells were plated in 24-well plates (4 × 10^4^/well) and treated with various concentrations of PT. After 24 h, cells were collected, mixed with an equal volume of trypan blue and then counted under the microscope.

### Colony formation assay

Huh-7 and SK-Hep-1 were seeded into 6-well plates (1000 cells/well) for 10 days in the presence of various concentrations of PT. Cells were then washed with phosphate-buffered saline (PBS), fixed with methanol and then subjected to 5% Giemsa staining.

### Annexin V/PI double staining

Muse Annexin V and a dead cell assay kit (EMD Millipore, Billerica, MA, USA) were used to analyse the apoptosis profile of PT-treated cells. The assay was performed according to the manufacturer’s protocol. Briefly, cells were grown in 6-well culture plates (4 × 10^5^/well) and treated with various concentrations of PT (0, 25, 50 and 100 μM) for 24 h. Afterwards, cells were trypsinised and resuspended in 100 μl of PBS. Then, 100 μl of Muse Annexin V and dead cell reagent were added to each tube, the content of each tube was thoroughly mixed and cells were subject to an additional incubation period without exposure to light. After incubation, the cells were analysed using the Muse cell analyser (EMD Millipore, Billerica, MA, USA).

### Quantification of acidic vesicular organelles

Acidic vesicular organelle (AVO) production was assessed using acridine orange (AO; Sigma-Aldrich, St. Louis, MO, USA) staining. Specifically, 4 × 10^5^ cells/well were seeded into 6-well plates and treated with various concentrations of PT. After 24 h, AO was added to the medium to reach a final concentration of 1 μg/ml, and the cells were further incubated at 37 °C for 30 min. AO-stained cells were then harvested, washed twice with PBS and analysed using a FACSCalibur flow cytometer (Becton, Dickinson, and Company, San Jose, CA, USA) with the Cell Quest Pro software.

### Quantification of ER expansion

ER expansion was assessed using ER-ID Red (Enzo Life Sciences Farmingdale, NY, USA) staining. Briefly, 4 × 10^5^ cells/well were seeded into 6-well plates and treated with various concentrations of PT for 24 h. Subsequently, the treated cells were harvested and stained with ER-ID Red reagent for 30 min at room temperature. A FACSCalibur flow cytometer (Becton, Dickinson, and Company, San Jose, CA, USA) and Cell Quest Pro software were used to analyse the stained cells.

### Detection of autophagy using transmission electron microscopy

After treatment, the cells were prefixed in 2.5% glutaraldehyde for 1.5 h and washed twice with 0.1 M PBS (pH 7.0). Two per cent of osmium tetroxide was further applied for 1 h for post-fixation. Dehydration was then performed in an ascending concentrations of ethanol. Finally, the cells were polymerised using Spurr resin at 72 °C for 12 h. Ultrathin sections obtained by UC7 ultramicrotome (Lecia Microsystems GmbH, Wetzlar, Germany) were stained with 1% uranyl acetate and 1% lead citrate and observed with Hitachi HT7700 transmission electron microscope (TEM) (Hitachi, Tokyo, Japan) at 100 kV accelerating voltage.

### siRNA transient transfection

Huh-7 cells were cultured on 6-cm dishes at 37 °C for 24 h. The cells were then incubated with Lipofectamine RNAiMAX reagent (Thermo Fisher Scientific, Waltham, MA, USA) and si-LC3, si-eIF2α or siATF4 (AllBio, Taichung, Taiwan). After 24 h, the cells were treated with PT (100 μM) for 24 h and analysed through Western blotting and immunofluorescence staining.

### Immunofluorescence staining

Cells were seeded into Lab-Tek 12-well chamber slides (Thermo Fisher Scientific, Waltham, MA, USA). After the indicated treatment, cells were fixed with 4% paraformaldehyde and permeabilised with 0.5% Triton X-100. Primary antibodies were then added to each well for overnight incubation at 4 °C. Subsequently, cells were incubated with DyLight-conjugated anti-rabbit or anti-mouse immunoglobulin G (IgG) and 4ʹ,6ʹ-diamidino-2-phenylindole (DAPI) for 1 h. Fluorescence images were captured using a confocal laser scanning microscope (Zeiss LSM 510 META, Jena, Germany).

### Chromatin immunoprecipitation assay

Huh-7 cells were seeded into a 10-cm dish and treated with or without 100 μM PT for 24 h. Cells were then crosslinked with 1% paraformaldehyde for 10 min at room temperature. Subsequently, 125 mM glycine was added, and cells were incubated at room temperature for 5 min. Chromatin harvested from cells was sonicated and immunoprecipitated with rabbit monoclonal antibody against ATF4 or with rabbit IgG. Chemical crosslinks were reversed through overnight incubation at 65 °C in the presence of 8 M NaCl. This was followed by the addition of RNase (20 mg/ml) for 1 h at 37 °C and proteinase K (10 mg/ml) for 2 h at 45 °C. After purification, DNA was dissolved in 20 μl of nuclease-free water. The chromatin immunoprecipitation (ChIP) primers used for the polymerase chain reaction (PCR) were as follows: LC3 (−1895/−1723)-F: 5′-CGGTTTCAAGCGATTCTC-3′ and LC3 (−1895/−1723)-R: 5′-ACTTTGGGAGGTCAAGGC-3′, LC3 (−528/−340)-F: 5′- GGAGGGGAAGGGATGGTCGG-3’ and LC3 (−528/−340)-R: 5′-CCTGAGGTGACG GTTGTGGG-3′. The PCR products were visualised with 2% agarose gels and ethidium bromide staining.

### Nuclear protein isolation

Cells were seeded into a 10-cm dish and treated with or without 100 μM of PT for 24 h. Nuclear protein was then isolated using a nuclear protein isolation kit (FIVEphoton Biochemicals, San Diego, CA, USA), according to the manufacturer’s instructions. Isolated proteins were then subjected to a Western blot analysis.

### Western blot analysis

Equal amounts of proteins (20 μg) isolated from cells were separated using sodium dodecyl sulfate-polyacrylamide gel electrophoresis and transferred to a polyvinylidene fluoride membrane. Membranes were then blocked with 5% nonfat dry milk buffer for 1 h at room temperature. After blocking, membranes were incubated with appropriate primary antibodies at 4 °C overnight, which was followed by incubation with secondary antibodies for 2 h at room temperature. The proteins were visualised using ECL reagents (EMD Millipore, Billerica, MA, USA), under the LAS-4000 mini luminescent image analyser (GE, PA, USA).

### Tumour xenograft in nude mice

For this process, 5 × 10^6^/0.1 ml of SK-Hep-1 cells were subcutaneously inoculated into the back of 4–5-week-old BALB/c female athymic mice purchased from the National Laboratory Animal Centre (Taipei, Taiwan). Treatments were initiated when tumours reached a mean group size of approximately 85 mm^3^. PT was orally administered to mice twice per week for 6 weeks. Tumour sizes and body weights were recorded every 7 days after 5 days of SK-Hep-1-cell injection, and tumour volume was measured using the following formula: length × width^2^ × 0.52. After treatment for 6 weeks, the tumour was removed, weighed and then prepared for immunohistochemical staining.

### Safety evaluation and histopathological analysis

To assess the safety profile in mice during treatment with PT, serum samples of mice were collected during scarification and subjected to analysis of biochemical parameters (aspartate aminotransferase (AST), alanine aminotransferase (ALT), blood urea nitrogen (BUN) and creatinine). Tissues from vital organs (heart, liver, kidney, spleen and lung) were sectioned, fixed in 10% formaldehyde, embedded in paraffin and finally subjected to haematoxylin and eosin staining and histopathological analysis under a light microscope.

### Statistical analysis

Differences in experimental results were statistically analysed using the one-way analysis of variance on SPSS (version 10.0). Results are expressed as mean ± standard deviation in triplicate, with a *p* value <0.05 or <0.01 considered statistically significant.

## Results

### PT inhibits the proliferation of HCC cells

**Fig. 1:**
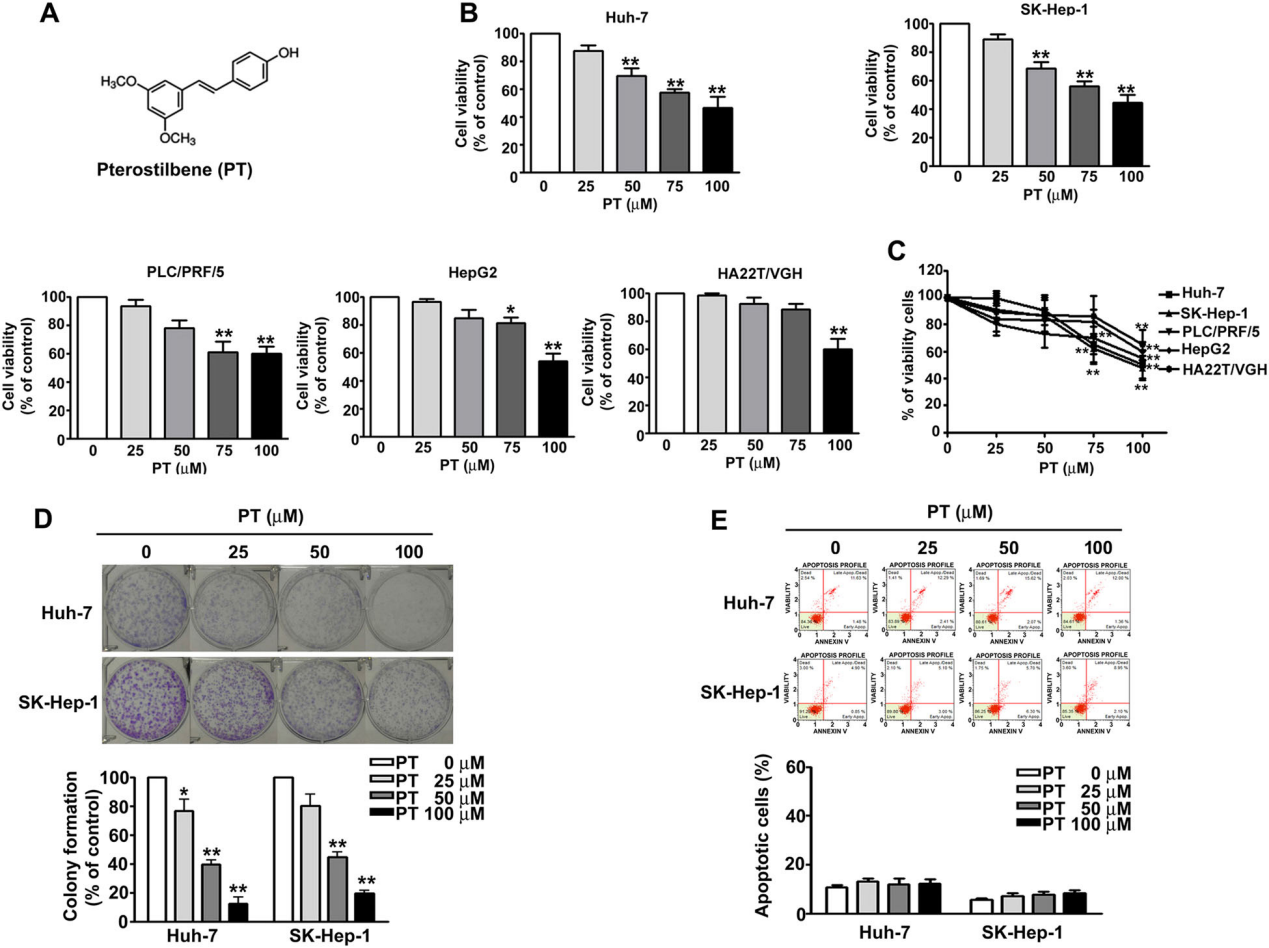
Pterostilbene (PT) inhibits the proliferation of hepatocellular carcinoma (HCC) cells. **a** Structure of PT. **b** Human HCC cell lines (Huh-7, SK-Hep-1, PLC/PRF/5, HA22T/VGH and HepG2) were treated with various concentrations of PT (0, 25, 50, 75 or 100 μM) for 24 h, followed by 3-(4,5-dimethylthiazol-2-yl)-2,5-diphenyl-tetrazolium bromide (MTT) assay. **c** Cell viability was determined through a trypan blue exclusion assay. **d** Colony formation assay was performed on HCC cells treated with PT for 7 days. **e** PT-treated cells were harvested and subjected to Annexin V and dead cell flow cytometry assay for the quantitative analysis of apoptosis. *p < 0.05, **p < 0.01 compared with controls. Data are presented as mean ± standard error for three experiments

To assess the effects of PT with the chemical structure depicted in Fig. 1a on cell viability, HCC cell lines (Huh-7, SK-Hep-1, PLC/PRF/5, HA22T/VGH and HepG2) were treated with various concentrations of PT (0, 25, 50, 75 and 100 μM) for 24 h, and cell viability was determined through MTT assay (Fig. 1b), trypan blue exclusion assay (Fig. 1c) and colony formation assay (Fig. 1d). Results demonstrated that PT at the highest concentration inhibited the growth of all studied cell lines; however, dose-dependent inhibition was observed in only Huh-7 and SK-Hep-1 cells. Annexin V/propidium iodide (PI) staining and flow cytometry were performed, and the results of those procedures demonstrated that PT did not induce apoptosis (Fig. 1e) or necrosis (Fig. S1) in Huh-7 and SK-Hep-1 cells. These results suggest that PT inhibits the growth of HCC cells in an apoptosis-independent manner.

### PT induces autophagy in HCC cells

**Fig. 2:**
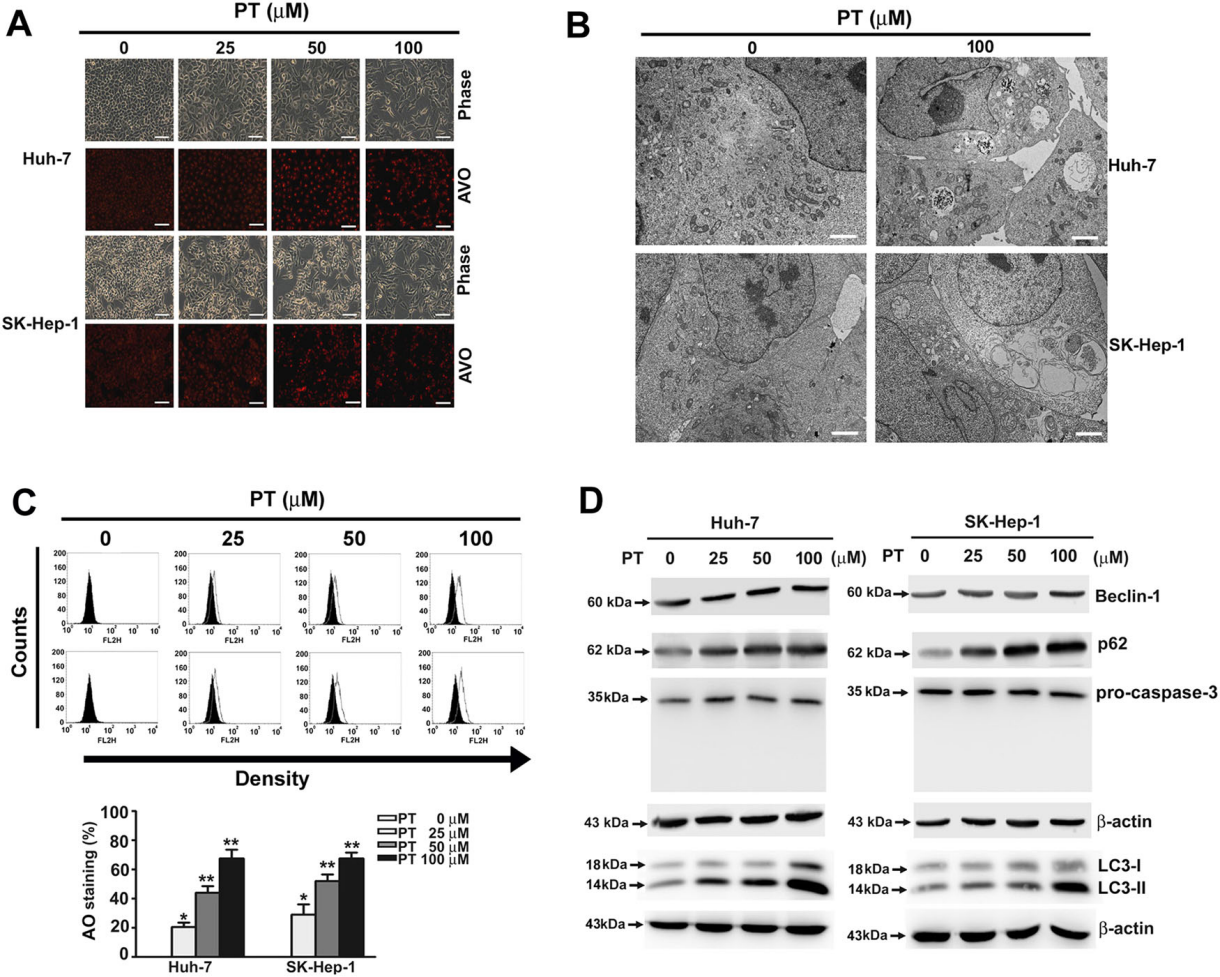
Pterostilbene (PT) induces autophagy in hepatocellular carcinoma (HCC) cells. **a** Huh-7 and SK-Hep-1 were treated with PT (0, 25, 50 and 100 μM) for 24 h, followed by acridine orange (AO) staining and visualisation under a fluorescence microscope. **b** Transmission electron microscope (TEM) image of the ultrastructure of SK-Hep-1 and Huh-7 cells treated with or without PT (100 μM) for 24 h. Scale bar: 5 μm. **c** Quantitative analysis of AO-stained cells using a flow cytometer. **d** Protein expression levels of Beclin-1, p62, LC3-II and pro-caspase-3 were assessed using Western blot analysis. β-Actin was employed as an internal control. **p < 0.01 compared with controls. *p < 0.05

Because of the finding of the lack of involvement of apoptosis in PT-induced cell death, our research then focused on autophagy to find the presence of numerous vacuoles in PT-treated cells, suggesting the involvement of autophagy in PT-induced cell death. AO staining confirmed that the development of AVOs in PT-treated Huh-7 and SK-Hep-1 cells increased in a dose-dependent manner (Fig. 2a). As depicted in Fig. 2b, autophagosomes were observed in PT-treated HCC cells using TEM. Flow cytometry was performed to confirm the finding of AVO formation discovered during AO staining (Fig. 2c). The results of Western blotting analysis suggested that PT increased the expression of Beclin-1, p62 and LC3-II in a dose-dependent manner, but the expression of pro-caspase-3 was not affected (Fig. 2d). Taken together, our results indicate that PT induces autophagy-dependent cell death without involving apoptosis in HCC cell lines.

### Autophagy is required for PT-induced cell death

**Fig. 3:**
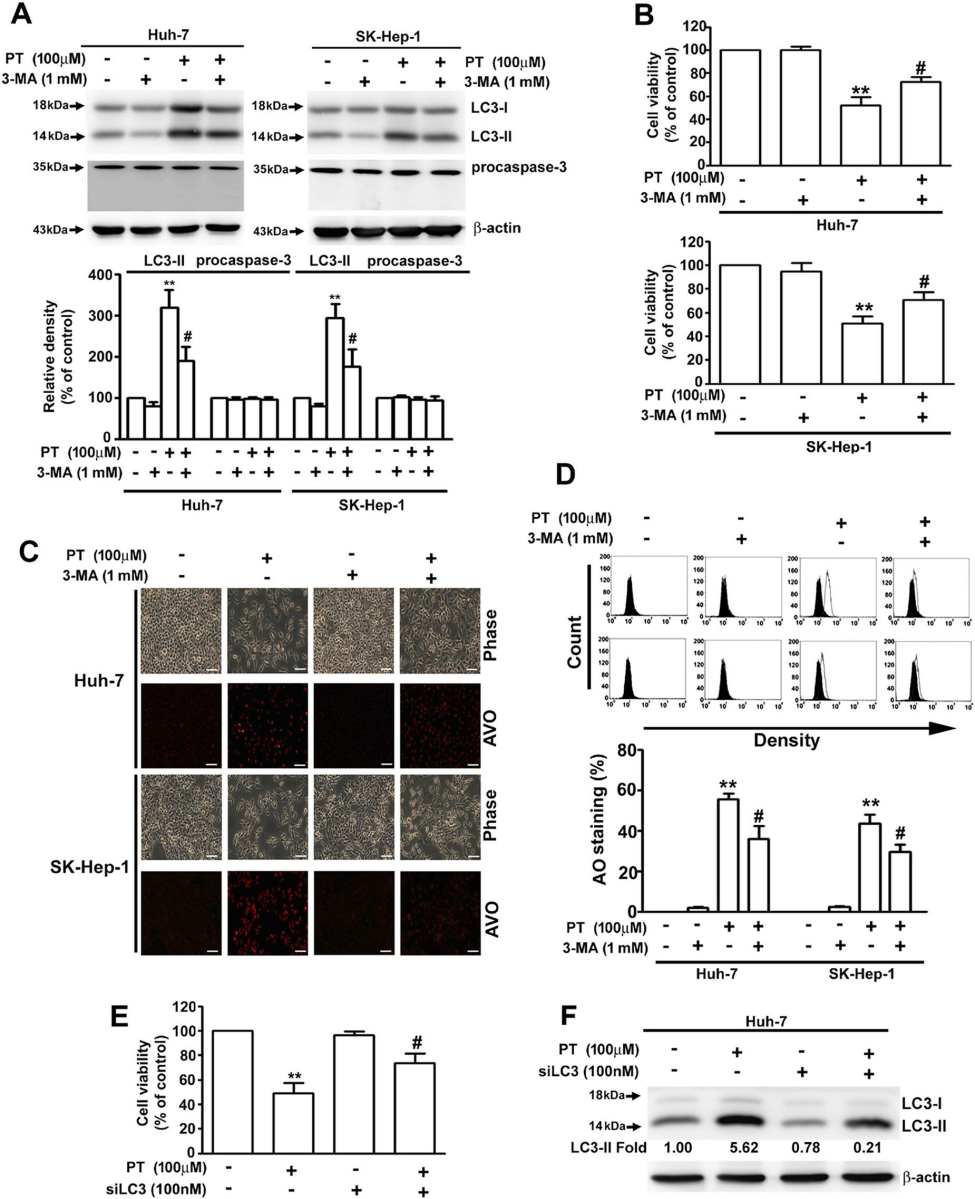
Autophagy is required for pterostilbene (PT)-induced cell death. **a** Hepatocellular carcinoma (HCC) cells were treated with PT (100 μM) in the presence or absence of the autophagy inhibitor 3-methyladenine (3-MA), and the protein expression levels were assessed using Western blot analysis. β-Actin was employed as an internal control. **b** Cell viability was measured using a 3-(4,5-dimethylthiazol-2-yl)-2,5-diphenyl-tetrazolium bromide (MTT) assay. **c** Formation of acidic vesicular organelles (AVOs) was observed under a fluorescence microscope. **d** Quantitative analysis of acridine orange (AO)-stained cells using a flow cytometer. **e** Huh-7 cells were treated with PT (100 μM) in the presence or absence of si-LC3 for 24 h, followed by Western blot analysis with β-actin serving as an internal control. **f** Cell viability was measured using an MTT assay. Data are presented as mean ± standard error for three experiments. **p < 0.01 compared with controls. ^#^*p* < 0.01 compared with PT treatment alone

To further assess the role of autophagy in PT-induced cytotoxicity, cells were pretreated with 3-MA, an autophagy inhibitor, prior to PT treatment. As illustrated in Fig. 3a, in the presence of 3-MA, the expression level of LC3-II was markedly decreased in PT-treated Huh-7 and SK-Hep-1 cells, but the expression of cleaved-caspase-3 was not affected. The results of MTT assay and AO staining revealed that 3-MA partially reduced the cell cytotoxicity (Fig. 3b) and decreased the production of AVOs of PT-treated cells (Fig. 3c, d). RNA interference attenuated the expression of LC3-II, and the viability of PT-treated Huh-7 cells was increased through the inhibition of LC3-II expression (Fig. 3e, f). These results suggest that PT exerts its cytotoxic effects partially through autophagic cell death in Huh-7 and SK-Hep-1 cells. To clarify the late-stage of autophagy in the PT-induced inhibition of cell viability, Huh-7 cells were pretreated with CQ (a late autophagy inhibitor) or a combination of E-64d (10 μM) and pepstatin A (10 μM), which are lysosomal protease inhibitors that inhibit late-stage autophagy. Pretreatment was followed by treatment with PT for 24 h. Neither CQ nor an E-64d/pepstatin A combination abolished the effect of PT to decrease cell viability and enhance p62 and LC3-II expression (Fig. S2). These results suggest that PT induces early-stage autophagy but is not involved in late-stage autophagy based on the autophagolysosomes in human HCC cells.

### PT induces ER stress in HCC cells

**Fig. 4:**
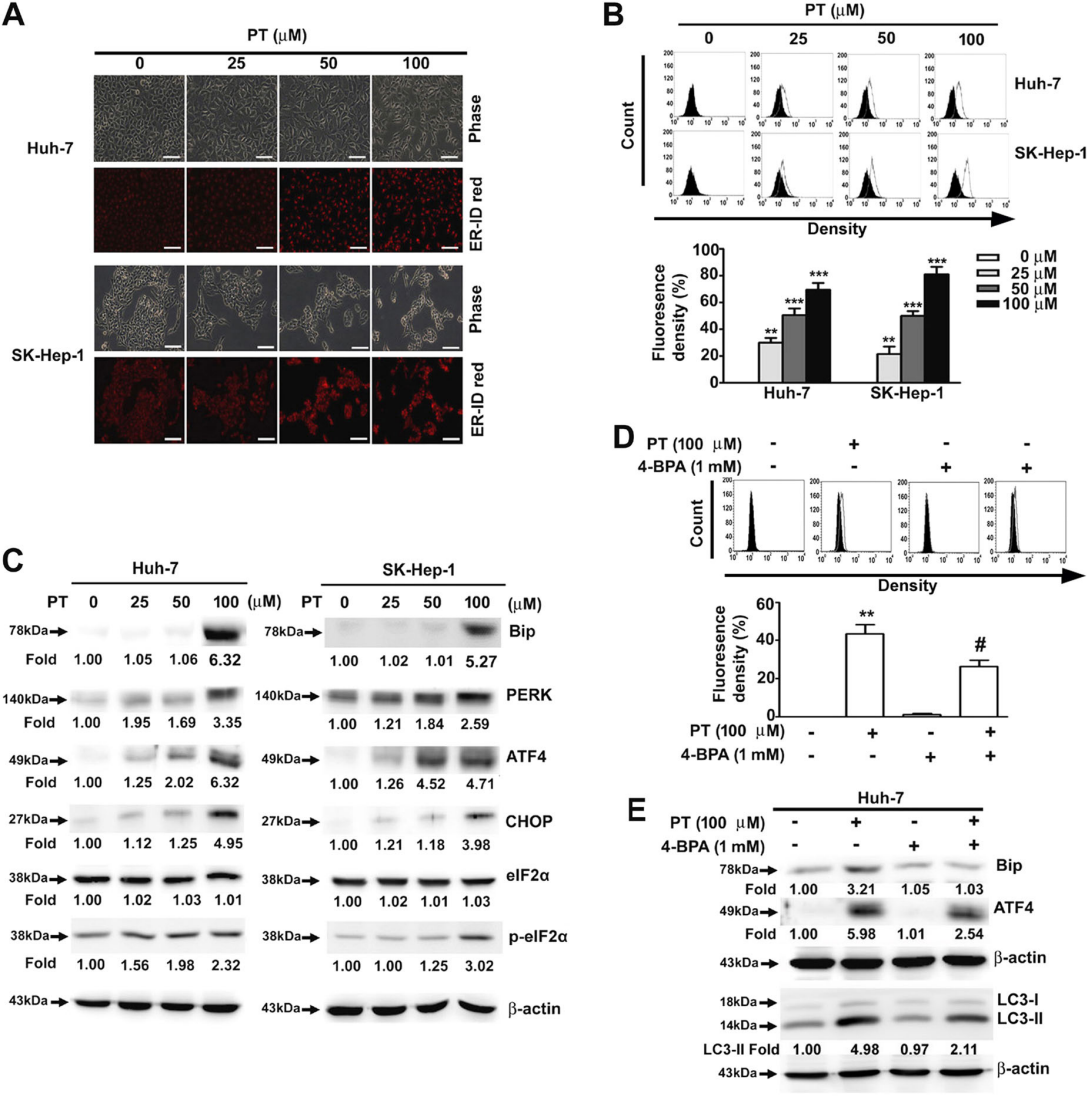
Pterostilbene (PT) induces endoplasmic reticulum (ER) stress in hepatocellular carcinoma (HCC) cells. Huh-7 and SK-Hep-1 cells were treated with various concentrations of PT for 24 h and then stained with ER-ID Red. **a** Morphological changes of ER were observed under a fluorescence microscope. **b** The intensity of fluorescence was quantified using a flow cytometer. **c** Western blotting was performed to investigate the expression levels of ER stress-related proteins. **d** Huh-7 cells were pretreated with or without 4-bisphenol A (4-BPA) (1 mM) for 2 h, followed by a treatment with PT (100 μM) for 24 h. The ER intensity of fluorescence was quantified using a flow cytometer. **e** Expression levels of Bip, activating transcription factor (ATF4) and LC3-II were determined through Western blotting analysis. β-Actin was employed as an internal control. Data are presented as mean ± standard error for three experiments. **p < 0.01 compared with controls. ^#^*p* < 0.01 compared with PT treatment alone

Studies have demonstrated that increased ER stress may lead to the activation of autophagy^19,20^, and ER expansion is one of the hallmarks of activated ER stress. Therefore, PT-treated cells were stained with an ER-specific fluorescence dye, ER-ID Red, to assess the morphological changes in the ER. As illustrated in Fig. 4a, b, PT dose-dependently increased the volume of ER and the ER-specific fluorescence intensity in HCC cells. To further investigate PT-induced ER stress, Western blotting was used to assess the expression of ER stress-related proteins and determine if PT significantly increased the expression levels of Bip, PERK, p-eIF2α, ATF4 and CHOP (Fig. 4c). Pretreatment with 4-bisphenol A, which inhibits ER stress by acting as a chemical chaperone, reduced the expansion of the ER lumen (Fig. 4d). Moreover, it not only reduced the expression of ER stress-related proteins but also the expression of the autophagy marker LC3-II (Fig. 4e). Collectively, these results indicate that PT induces ER stress in HCC cells.

### PT induces autophagic cell death through the ATF4/LC3 pathway

**Fig. 5:**
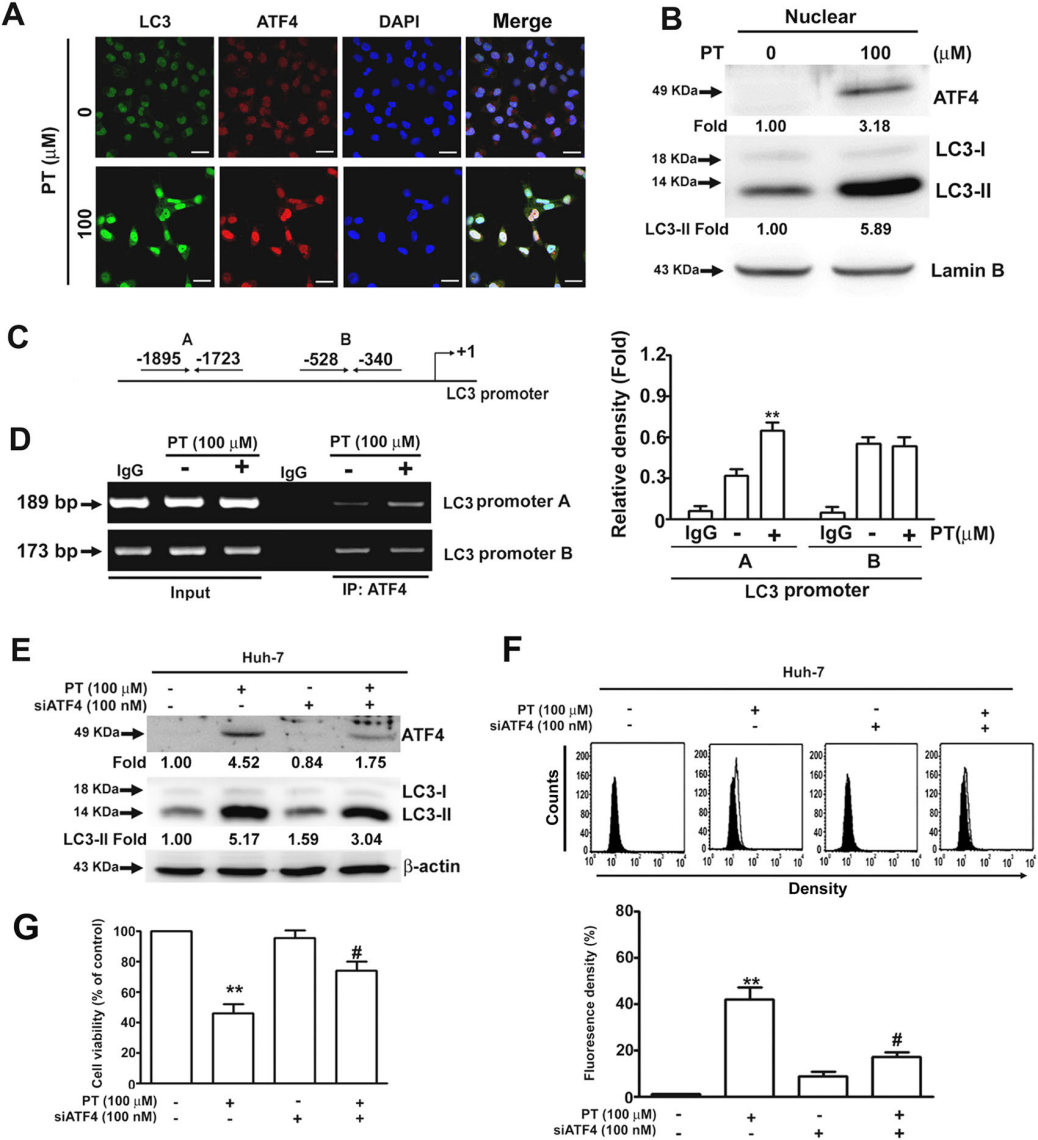
Pterostilbene (PT) induces activating transcription factor-4 (ATF4) binding to the LC3 promoter, which mediates cell death. **a** Huh-7 cells treated with PT (100 μM) for 24 h were fixed; permeabilised; stained with anti-ATF4 antibody, anti-LC3 antibody and 4ʹ,6ʹ-diamidino-2-phenylindole (DAPI); and then visualised under a confocal microscope. **b** Nuclear proteins were isolated and assessed using Western blot analysis with Lamin serving as an internal control. **c** The binding sites of ATF4 transcription factor on the LC3 promoter are labelled. **d** Chromatin immunoprecipitation (ChIP) assay using anti-ATF4 antibodies was performed with DNA samples extracted from PT-treated Huh-7 cells. Input samples were used as positive controls. **e** Huh-7 cells were treated with PT (100 μM) in the presence or absence of siATF4 for 24 h. The protein expression levels were assessed using Western blot analysis. β-Actin served as an internal control. **f** Quantitative analysis of acridine orange (AO)-stained cells was performed using a flow cytometer. **g** Cell viability was measured using a 3-(4,5-dimethylthiazol-2-yl)-2,5-diphenyl-tetrazolium bromide (MTT) assay. Data are presented as mean ± standard error for three experiments. **p < 0.01 compared with controls. ^#^*p* < 0.01 compared with PT treatment alone

A study demonstrated that ATF4 is essential for ER-induced autophagy^21^. Our results from immunofluorescence staining and Western blotting indicated that PT induced a nuclear translocation of ATF4 in Huh-7 cells (Fig. 5a, b). On the basis of another study that determined that ATF4 can bind to the LC3 promoter^22^ and the putative ATF4-binding sequences presented in Fig. 5c, this study undertook a ChIP analysis, the results of which indicated that PT increased ATF4’s interaction only with the −1895 to −1723 region of the LC3 promoter, but had no effect on the −528 to −340 region (Fig. 5d). We next examined whether the knockdown of ATF4 affected PT-induced cell death and found that ATF4 silencing markedly reduced the expression of LC3-II (Fig. 5e). Furthermore, the results of flow cytometry and MTT assays revealed that the silencing of ATF4 significantly reduced the production of AVOs (Fig. 5f) and cell death (Fig. 5g) in PT-treated Huh-7 cells. Both assays indicated that ATF4 is essential for PT-induced autophagy.

### eIF2α is essential for PT-induced autophagy

**Fig. 6:**
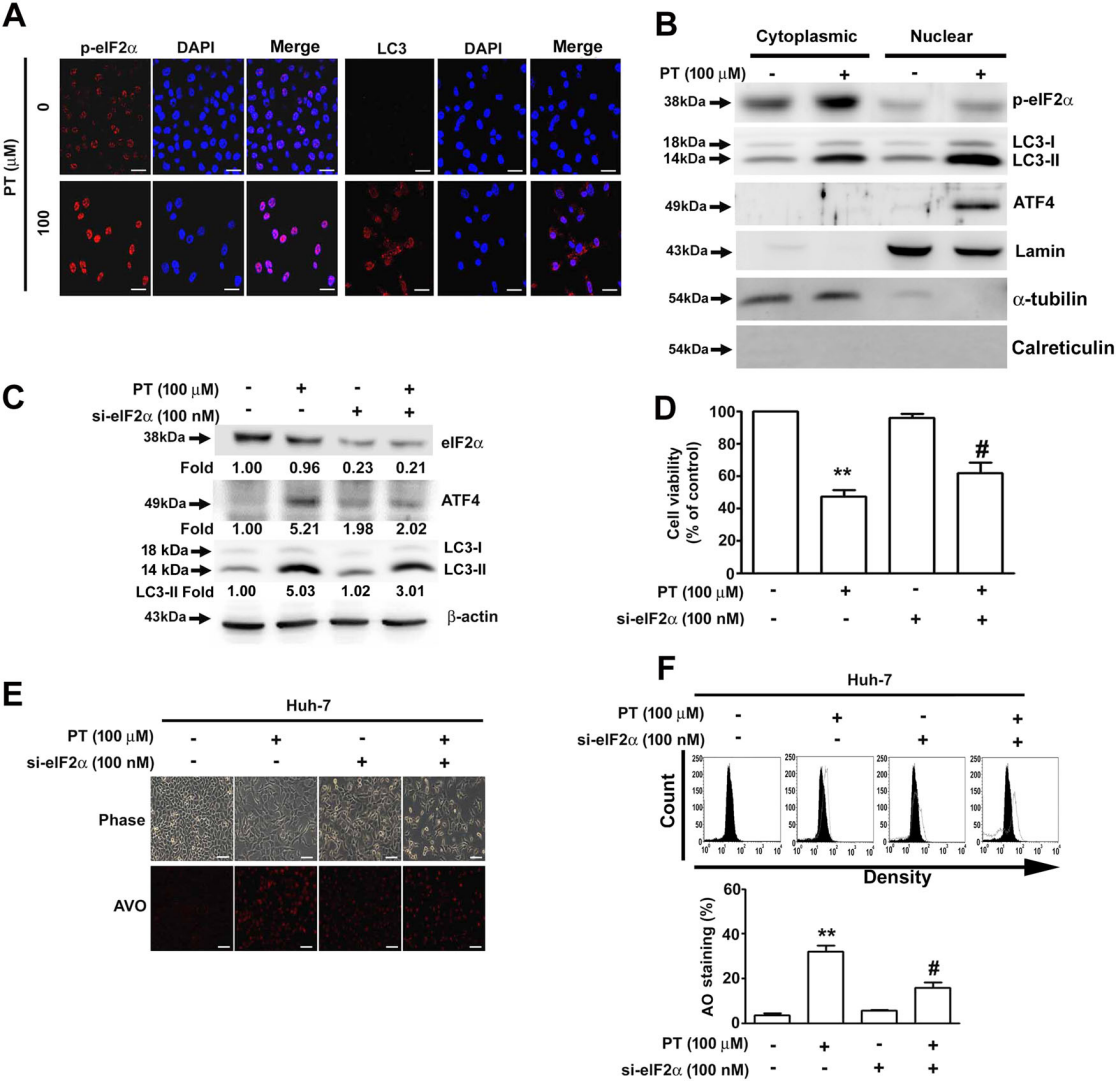
Eukaryotic initiation factor 2α (eIF2α) is essential for pterostilbene (PT)-induced autophagy. **a** Huh-7 cells treated with PT for 24 h were fixed; permeabilised; stained with anti-p-eIF2α antibody, anti-LC3 antibody and 4ʹ,6ʹ-diamidino-2-phenylindole (DAPI); and then visualised under a confocal microscope. **b** Cytoplasmic and nuclear proteins were isolated and the p-eIF2α, LC3-II and ATF4 protein expression levels were measured using Western blot analysis. Lamin B: nuclear marker; α-tubulin: cytoplasmic marker; and calreticulin: ER marker. **c** Huh-7 cells were treated with PT (100 μM) in the presence or absence of si-eIF2α for 24 h. The protein expression level was assessed using Western blot analysis with β-actin serving as an internal control. **d** Cell viability was measured using a 3-(4,5-dimethylthiazol-2-yl)-2,5-diphenyl-tetrazolium bromide (MTT) assay. **e** Formation of acidic vesicular organelle (AVOs) was visualised through acridine orange (AO) staining and observed under a fluorescence microscope. **f** Quantification was performed using a flow cytometer. Data are presented as mean ± standard error for three experiments. **p < 0.01 compared with controls. ^#^*p* < 0.01 compared with PT treatment alone

Because the PERK/eIF2α/ATF4 pathway is strongly linked to autophagy^23^, we sought to verify the role of eIF2α in PT-induced cell death, using immunofluorescence staining to identify increased expression of p-eIF2α and LC3 (Fig. 6a) and upregulation of p-eIF2α, LC3-II and ATF4 proteins in the nuclei of PT-treated cells (Fig. 6b). Knockdown of eIF2α significantly reduced the expression of ATF4 and LC3-II (Fig. 6c) and increased cell viability (Fig. 6d) in PT-treated cells, and the increased production of AVOs was also attenuated by eIF2α knockdown in PT-treated Huh-7 cells (Fig. 6e, f), indicating that eIF2α plays a crucial role in PT-induced cell death.

### Enhanced phosphorylation of eIF2α increased PT-induced cell death in an autophagy-dependent manner

**Fig. 7:**
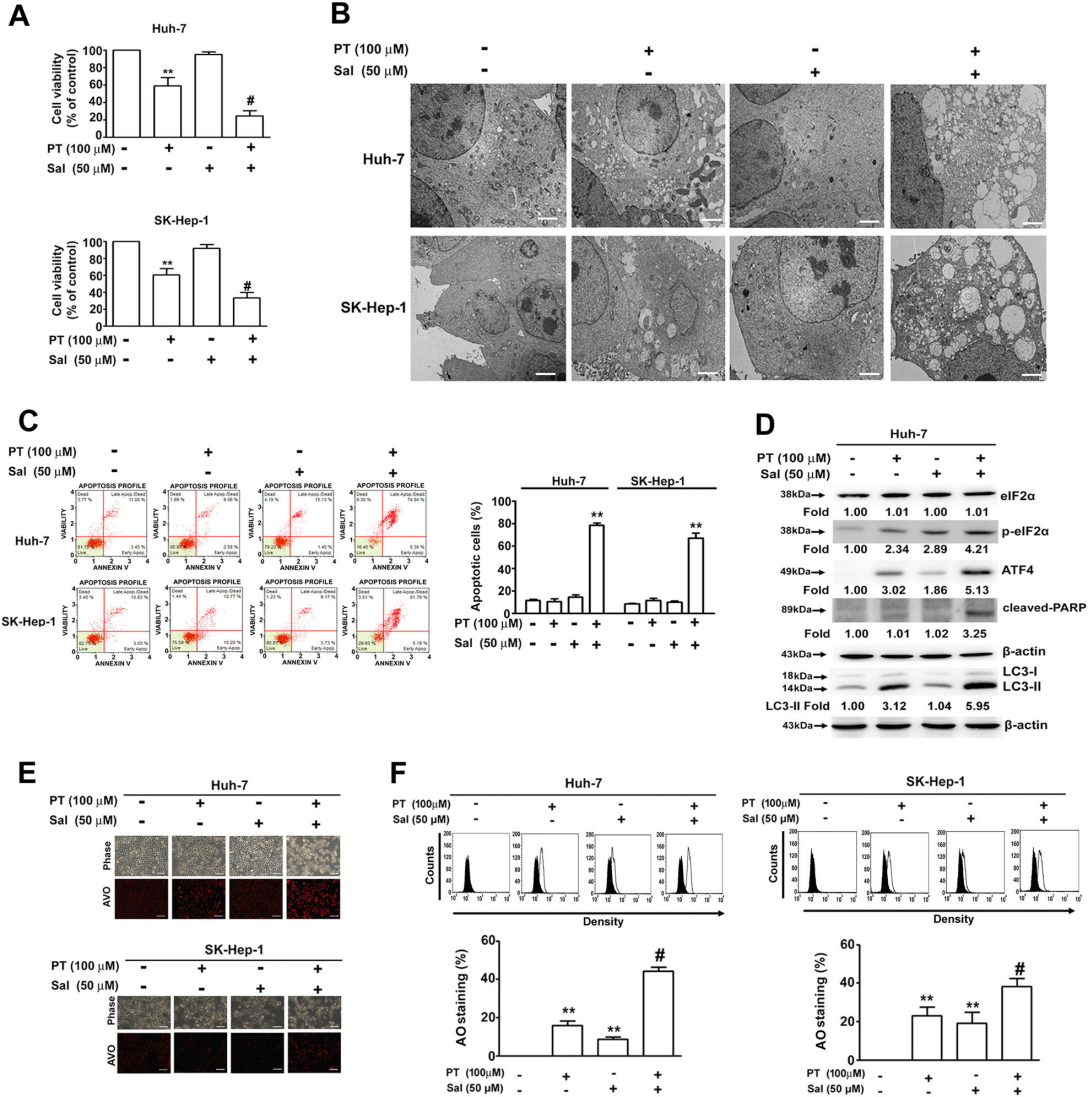
Enhanced phosphorylation of eukaryotic initiation factor 2α (eIF2α) increased pterostilbene (PT)-induced cell death. Huh-7 and SK-Hep-1 cells were treated with PT (100 μM) in the presence or absence of salubrinal (Sal) (50 μM) for 24 h. **a** Cell viability was measured using a 3-(4,5-dimethylthiazol-2-yl)-2,5-diphenyl-tetrazolium bromide (MTT) assay. **b** Representative transmission electron micrographs of the ultrastructure of SK-Hep-1 and Huh-7 cells treated with PT (100 μM) in the presence or absence of Sal (50 μM) for 24 h. Scale bar = 5 μm. **c** For the quantitative analysis of apoptosis, cells were harvested and subjected to Annexin V and dead cell flow cytometry assays. **d** Protein expression levels were assessed using a Western blot analysis. β-Actin served as an internal control. **e** The formation of acidic vesicular organelles (AVOs) was visualised through acridine orange (AO) staining and observed under a fluorescence microscope and **f** quantified using a flow cytometer. Data are presented as mean ± standard error for three experiments. **p < 0.01 compared with controls. ^#^*p* < 0.01 compared with PT treatment alone

Because ATF4 expression is mediated by the phosphorylation of eIF2α^24^, an eIF2α phosphatase inhibitor (Sal) was used together with PT to evaluate the role of eIF2α in PT-induced cell death. We determined that the combination of PT and Sal may increase cytotoxicity (verified through a MTT assay; Fig. 7a), induce autophagy (verified through a TEM assay; Fig. 7b) and increase apoptosis (verified by Annexin V and dead cell assays, Fig. 7c) in HCC cells. Furthermore, we determined that the expression of p-eIF2α, ATF4, LC3-II and cleaved-PARP was increased through this combination treatment (Fig. 7d), as was an increased production of AVOs (Fig. 7e, f). Taken together, our results suggest that the enhanced phosphorylation of eIF2α increases autophagy-dependent cell death induced by PT.

### PT suppressed the growth of SK-Hep-1 xenografts in vivo and safety evaluation

**Fig. 8:**
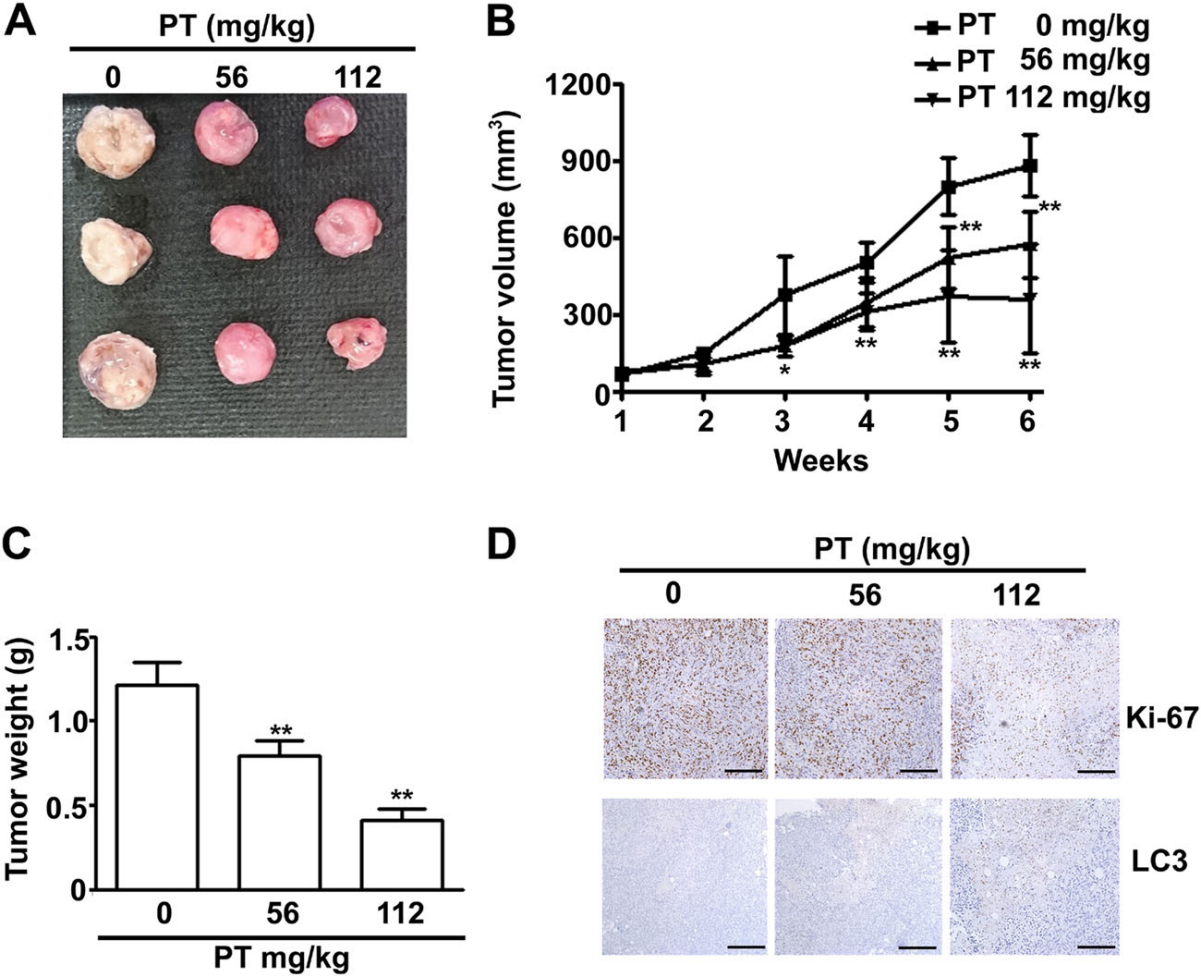
Pterostilbene (PT) suppressed the growth of SK-Hep-1 xenografts in vivo. BALB/c nu/nu mice were subcutaneously injected with SK-Hep-1 cells. After a tumour establishment period (1 week), the nude mice were orally given PT (56, 112 mg/kg) or dimethyl sulfoxide (DMSO) twice per week. **a** Image of representative tumours, **b** average tumour volume and **c** average tumour weight. **d** Tumour tissues were examined through immunohistochemistry staining. *p < 0.05, **p < 0.01 compared with controls. Scale bar: 100 μm

**Fig. 9:**
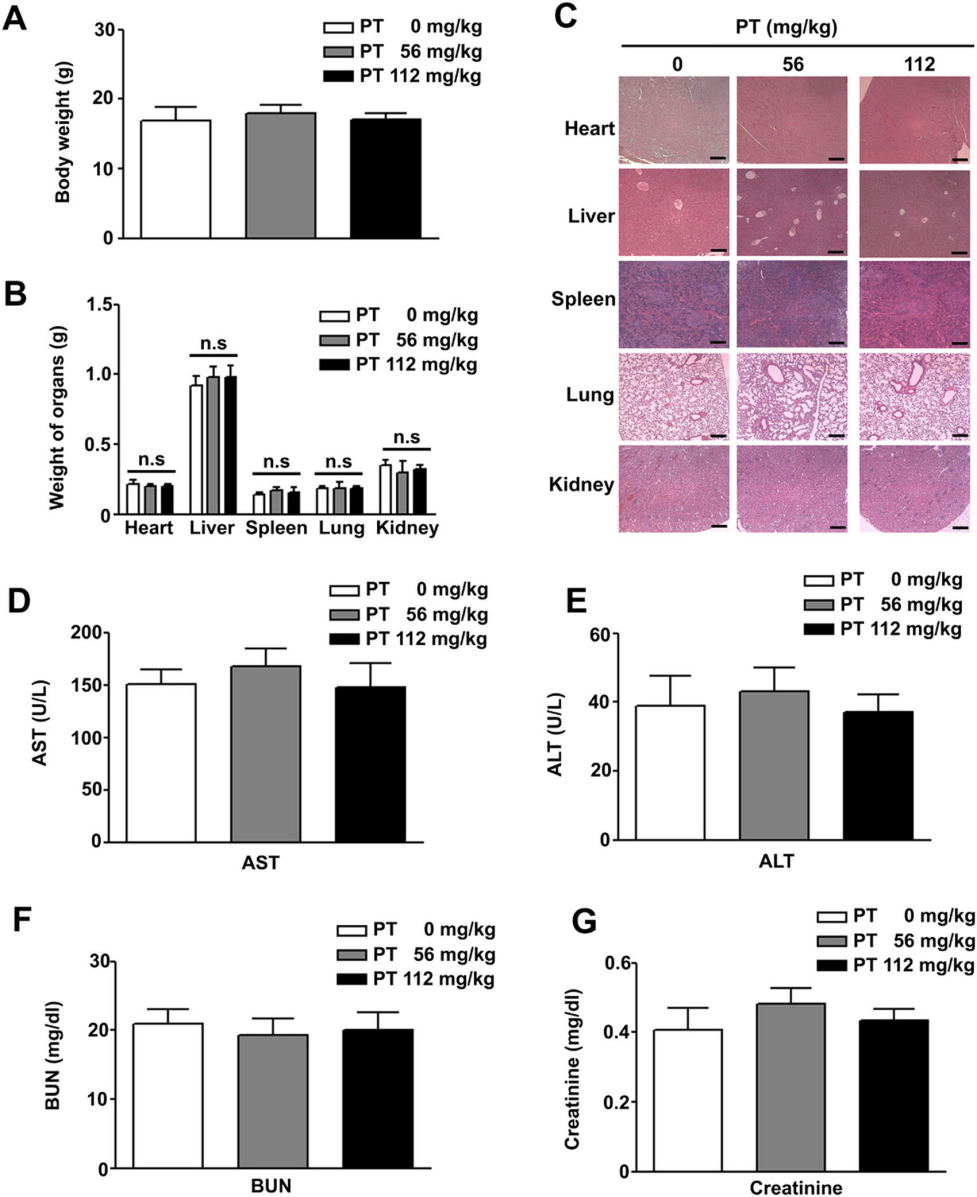
Tissue toxicity of pterostilbene (PT) in mice in vivo. PT was administered orally at a dose of 56 and 112 mg/kg twice per week for 6 weeks. **a**, **b** The body weight and organ weights of each group were measured. **c** Histopathological changes in organs (heart, liver, spleen, lung and kidney) were examined using haematoxylin and eosin staining. **d**–**g** Parameters of aspartate aminotransferase (AST), alanine aminotransferase (ALT), blood urea nitrogen (BUN) and creatinine from each group were measured. Scale bar: 100 μm. NS: not significant

After demonstrating the antitumour potential of PT against HCC cells, we next examined the effect of PT on SK-Hep-1 xenografts in vivo. As illustrated in Fig. 8a, oral administration of PT (56 or 112 mg/kg, twice per week) significantly inhibited tumour growth (Fig. 8b) and tumour weight of an SK-Hep-1 (Fig. 8c) xenograft. We also sought to determine the effects of PT treatment on the expression of Ki-67 (tumour proliferation marker) and LC3 (autophagy marker) using an immunohistochemistry analysis. The results indicated that the expression of the Ki-67 protein was substantially decreased and the expression of LC3 was increased in the PT treatment group compared with the results for the control group (Fig. 8d). No significant difference in organ weight and body weight was measured between the PT treatment group and the control group (Fig. 9a, b). Moreover, haematoxylin and eosin staining assay failed to indicate any obvious damage to the lung, liver, heart, kidney and spleen tissues of the PT and control groups (Fig. 9c). Neither blood biochemical analyses nor a histopathological examination revealed significant differences in serum AST and ALT levels between the PT treatment group and the control group (Fig. 9d, e). Furthermore, no obvious changes in serum BUN and creatinine levels, crucial indicators of kidney damage and nephrotoxicity, were apparent in the PT treatment group compared with the control group (Fig. 9f, g). Taken together, these results indicate that PT administered at the tested concentrations did not lead to any clinically significant side effects in mice.

## Discussion

Numerous studies have reported the antitumour and antioxidant activity and apoptosis- and autophagy-inducing property of PT in various tumour cells in vitro and in vivo^3^. PT suppresses the 12-*O*-tetradecanoylphorbol-13-acetate-induced invasion of HepG2 cells through the inhibition of mitogen-activated protein kinase and phosphoinositide 3-kinase pathways^8^ and inhibits breast cancer metastasis through the microRNA-mediated downregulation of the epithelial-to-mesenchymal transition^25^. Hsieh et al.^5^ discovered that PT induced both apoptosis and autophagy in lung cancer cells and that the inhibition of autophagy enhanced the apoptosis-inducing effect of PT. However, Ko et al.^26^ determined that the inhibition of autophagy hindered the apoptosis-inducing effect of PT, indicating that a complex relationship exists between PT-induced autophagy and apoptosis. In agreement with these studies, our data demonstrated that PT effectively inhibited the cell viability and colony formation ability of HCC cells through the induction of autophagy and ER stress and that the inhibition of autophagy attenuated the antitumour effect of PT in HCC cells. Thus, PT may act as a potential antitumour reagent in HCC cells.

Some studies have reported that ER stress is involved in PT-induced apoptosis in oesophageal cancer cells^12^ and non-small-cell lung cancer cells^27^. Moreover, PT induces apoptosis through p53/reactive oxygen species-mediated apoptosis in HepG2 cells^28^ and the alteration of p53 acetylation^29^, and retains the expression of the phosphatase and tensin homologue through the inhibition of microRNA-19a expression to inhibit the growth of SMMC7721 cells^30^. Other studies have also demonstrated that PT can induce apoptosis through cell cycle arrest in acute myeloid leukaemia cells^31^ and B cell lymphoma cells^32^. Regarding the particular role of ER stress in PT’s inhibitory effect on cell proliferation in HCC cells, UPR-dependent cell cycle arrest is considered an opportunity for cells to restore cellular homeostasis^33^. In this study, we determined that PT inhibited cell proliferation in HCC cells without inducing apoptosis but significantly induced the cell cycle arrest during the G0/G1 and S phase in HCC cells (Fig. S3). Similarly, studies have reported that the induction of cell cycle arrest may effectively control tumour cell proliferation^34^, treatment with resveratrol may induce cell cycle arrest at the S phase through activation of Cdc2 expression in human ovarian OVCAR-3 cells^35^ and pactamycin analogues may induce S-phase arrest and consequently suppress the proliferation of HNSCC cells through the p53-dependent pathway^36^. Numerous other studies have also demonstrated that a natural product, such as cannabisin B^37^ or rhein^38^, may result in similar cell cycle arrest at the S phase in other tumour cell types. The interaction between autophagy and S-phase arrest modulated by PT treatment in HCC cells should be studied further.

LC3 is a key autophagy regulator that is distributed abundantly in the nucleus and cytoplasm^39^. We have observed that PT may lead to a significant accumulation of LC3 expression in the nuclei, which suggests the possibility of nuclear LC3 having a key role in PT-induced autophagy. Huang et al.^40^ suggested that nuclear-localised Sirt1 becomes activated and deacetylated LC3 is expressed during cellular starvation. Deacetylated LC3 protein was translocated into the cytoplasm and interacted with autophagy-related protein 7 to facilitate the formation of autophagosomes, indicating that the deacetylation of nuclear LC3 plays a pivotal role in regulating the formation of autophagosomes^40^. Consistently, our findings revealed that PT increased the expression of nuclear LC3 and is involved in the formation of autophagosomes. In addition, PT may retard sepsis-induced liver injury by inhibiting inflammatory response and apoptosis through its association with Sirt1, which becomes activated, and deacetylated FoxO1, p53 and nuclear factor-κB are expressed^41^. Based on these evidences, further study is recommended to verify whether PT activates nuclear LC3 through Sirt1-induced deacetylation in HCC cells.

We proposed that PERK/eIF2α may be involved in the PT-induced cell cycle arrest of HCC cells. Numerous studies have documented that the downregulation of cyclin D1, harbouring targeted deletion of PERK expression, is sufficient to trigger cell cycle arrest in the G0/G1 phase during UPR activation, which is also associated with the PERK regulation of cyclin D1 translation^42^. A recent study contended that the inhibition of PERK and GCN2 is necessary and adequate for the loss of cyclin D1, precludes eIF2α phosphorylation and promotes cell cycle arrest after the induction of the UPR-signalling pathway^43^. In the present study, the inhibitory effects of PT may have been involved in cell cycle arrest through PERK-eIF2α activation. Therefore, whether the PERK-eIF2α pathway is involved in the cell cycle arrest of PT-mediated cell death via UPR activation in HCC cells requires further investigation.

The activation of ER stress is initially intended to restore ER homeostasis for cell survival; however, prolonged ER stress eventually causes cell death^44^. Our results indicated that PT induced the expansion of the ER lumen and increased the expression of ER stress-related genes as well as induced the nuclear translocation of ATF4 in HCC cells. The transcriptional control of LC3 is a fundamental factor in its regulation of apoptosis and autophagy. A relevant study suggested that the promoter of LC3 contains a CRE-like docking sequence of ATF4^22^, and our results indicated that PT significantly affected the phosphorylation of eIF2α in PT-treated cells and then directly affected the association between the ATF4 transcription factor and the promoters of LC3. Recent reports have suggested that CHOP plays a vital role in autophagy and apoptosis^45^, and it is widely acknowledged that the docking sequence for ATF4 is located within the CHOP promoters^46,47^. In our results, PT also increased the expression of CHOP in HCC cells, possibly because of the increased binding of ATF4 to the CHOP promoter after PT treatment. Thus, further experiments are required to investigate the molecular mechanism and the role of CHOP in the antitumour activity of PT in HCC cells.

The phosphorylation of eIF2α, a component of integrated responses to ER stress, results in the global attenuation of protein synthesis and enables the correct translation of ATF4 and other ER stress-related genes^15^. Sal, a selective inhibitor of eIF2α phosphatase, attenuates global translation and increases the activation of the UPR pathway, thus inhibiting ER stress-induced apoptosis^48^. A recent study determined that the eIF2α-related dephosphorylation of Sal in response to tumour necrosis factor-related apoptosis-inducing ligand treatment facilitates increased CHOP expression, the activation of eIF2α phosphorylation, caspase activation and, consequently, the induction of apoptosis^49^. Other studies have reported that Sal enhanced doxorubicin-mediated apoptosis in breast cancer cells^50^ and neuroblastoma cells^51^. In this study, we posit that the phosphorylation of eIF2α is responsible for the stimulation of PT-induced autophagic cell death induced by Sal.

The oral administration of PT has been demonstrated to be an effective in vivo delivery method, achieving a higher plasma concentration than resveratrol^52^. Furthermore, the dietary supplementation of PT inhibited the growth of xenograft tumours of MDA-MB-468 triple-negative breast cancer cells^53^. Similarly, both low (56 mg/kg) and high doses (112 mg/kg) of PT inhibited the growth of xenograft tumours, with a human equivalent dose of approximately 4.6 and 9.1 mg/kg, respectively, according to Federal Drug Administration guidelines. In addition, a histological analysis of the major organs did not reveal tissue damage, and a biochemical analysis did not reveal hepatic or renal damage in both low- and high-dose groups. Moreover, in a recent clinical trial evaluating the effect of PT on cholesterol, blood pressure and oxidative stress, the oral administration of 250 mg of PT per day was determined to be a safe dose and had a blood pressure-lowering effect^54^. This further supports the contention that these doses of PT elicit no systemic toxicity in normal tissue, suggesting that PT inhibits tumour growth in vitro with an excellent safety profile.

In conclusion, our results indicate that treatment with PT in combination with Sal enhances autophagy-dependent cell death through the involvement of the p-eIF2α/ATF4/LC3 pathway and may represent a novel anticancer agent for HCC treatment.

## Supplementary information

SUPPLEMENTAL DATA
