# Supplementary material for: Inhibition of eIF2α dephosphorylation accelerates pterostilbene-induced cell death in human hepatocellular carcinoma cells in an ER stress and autophagy-dependent manner
Source: Cell Death Dis. 2019 May 28;10(6):418. doi: 10.1038/s41419-019-1639-5 (PMC6538697; doi:10.1038/s41419-019-1639-5)
Supplement: Supplementary file 1 — SUPPLEMENTAL DATA [file 41419_2019_1639_MOESM1_ESM.docx]

Inhibition of eIF2α dephosphorylation accelerates Pterostilbene-induced cell death in human hepatocellular carcinoma cells in an ER stress and autophagy-dependent manner

**Supplemental Figure-1**

**
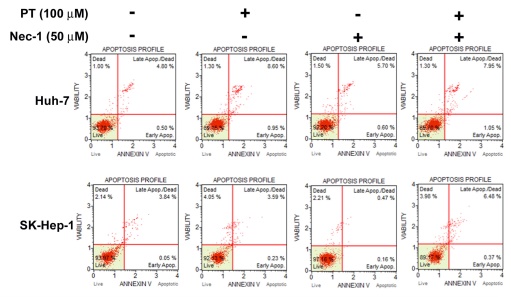
**

**Supplementary Figure 1.** Huh-7 and SK-Hep-1 cells were treated with PT (100 μM) in the presence or absence of the necrosis inhibitor Nec-1 (50 μM), quantitative analysis of Annexin V/PI-stained cells using a flow cytometer.

**Supplemental Figure-2**


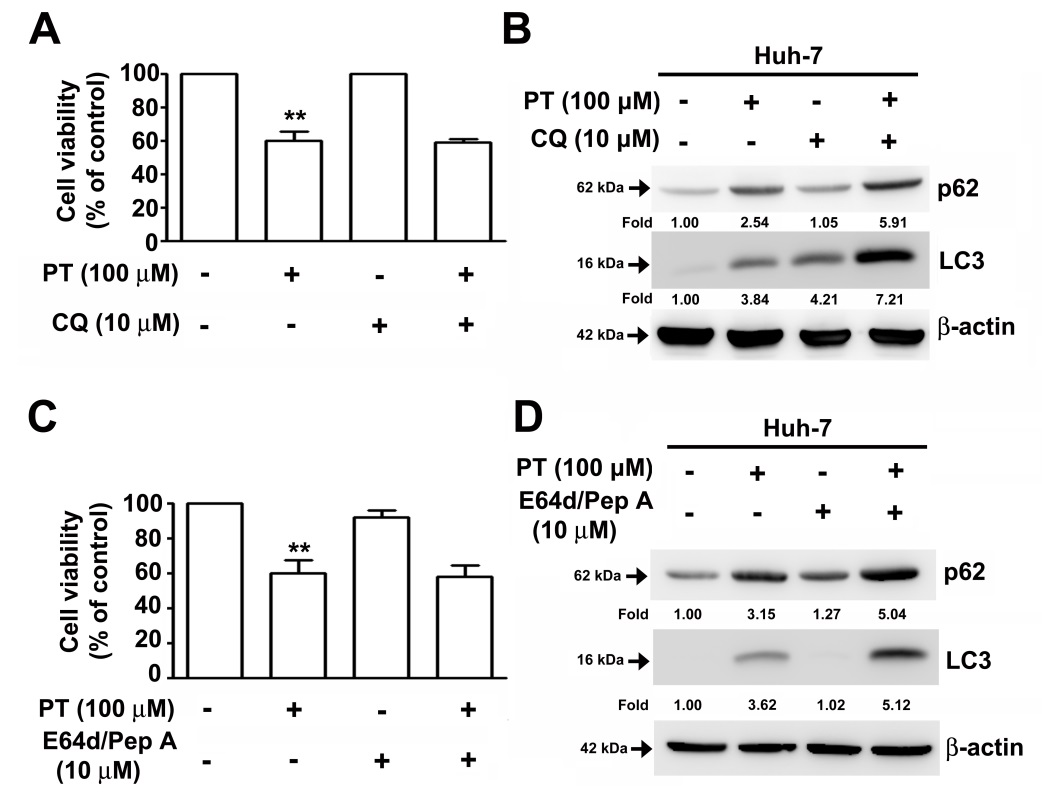


**Supplemental Figure-2** Effect of CQ or combined of E-64d/pepstatin A in PT treated Huh-7 cells. Huh-7 cells were pretreated with CQ or combined of E-64d/pepstatin A for 2 h, then added the PT (100 μM) for 22 h. **a, c.** Cell viability was measured using an MTT assay. **b, d**. The p62 and LC3 protein expression levels were assessed using a western blot analysis. β-actin served as an internal control. Data are presented as the mean±SE of at least three independent experiments. **p < 0.01 compared with controls.

**Supplemental Figure-3**


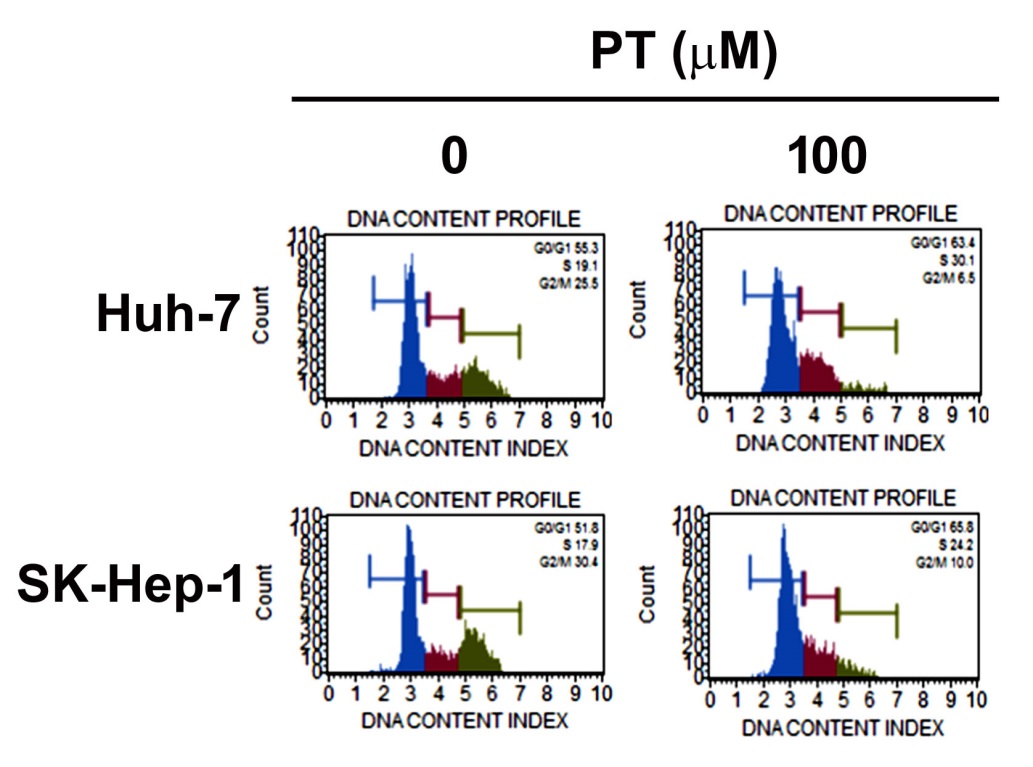


**Supplemental Figure-3** Effect of PT on cell cycle regulation in Huh-7 and SK-Hep-1 cells. Huh-7 and SK-Hep-1 cells were treated without or with PT (100 μM) and cell cycle progression was measured by flow cytometry.
